# Possible causes of seizure after spine surgery

**DOI:** 10.4103/1817-1745.66661

**Published:** 2010

**Authors:** Zohreh Habibi, Farideh Nejat, Mostafa El Khashab

**Affiliations:** Department of Neurosurgery, Imam Khomeini Hospital, Tehran, Iran; 1 Children’s Hospital Medical Center, Tehran University of Medical Sciences, Tehran, Iran; 2 Hackensack University of Medical Center, Hackensack, NJ, USA

**Keywords:** Lipomyelomeningocele, seizure, spinal-induced

## Abstract

Seizure after laminectomy for spinal procedure is very rare and has not been reported after lipomyelomeningocele surgery beforehand. Here, two cases of seizure following laminectomy for lipomyelomeningocele are reported. The exact etiology of the event is unknown but anesthetic material, pneumocephalus, intracranial hypotension subsequent to cerebrospinal fluid leakage after spinal procedures, spinal-induced seizure and the potential toxic effect of fat molecules could be considered.

## Introduction

Generalized seizure after laminectomy is very rare and, to our knowledge, has not been reported after lipomyelomeningocele surgery so far. Here, two cases of seizure following laminectomy for lumbar and lumbosacral lipomyelomeningocele are reported, and different aspects regarding the potential etiology of the event have been discussed.

## Case Report

Among all 107 cases of lipomyelomeningocele who underwent surgery in our center between 2000 and 2008, two patients developed generalized seizure postoperatively. One of them was a 7-month-old boy with lumbosacral transitional-type lipomyelomeningocele and the other was a 9-month-old boy with dorsal-type lipomyelomeningocele in the lumbar area. On admission, both patients had normal motor function but decreased anal sphincter tone and neurogenic bladder confirmed by urodynamic study. None of the children had a previous history of seizure. The patients had neither siblings with the same problem nor history of seizure in their families.

Preoperative laboratory tests were in the normal range. Standard surgical and anesthetic approach was performed in both patients. The patients were premedicated with fentanyl (1 μg/kg). Anesthesia was induced with inhalation of halothane and intravenous thiopental (5 mg/kg) and maintained with halothane at 1.5 minimum alveolar anesthetic concentration and N2O 50%. The patients then turned prone and standard surgery for lipomyelomeningocele was performed, including lipoma resection, releasing nerve roots, cord untethering, placode pial suturing and repairing dural and facial defects.

**Figure 1:**
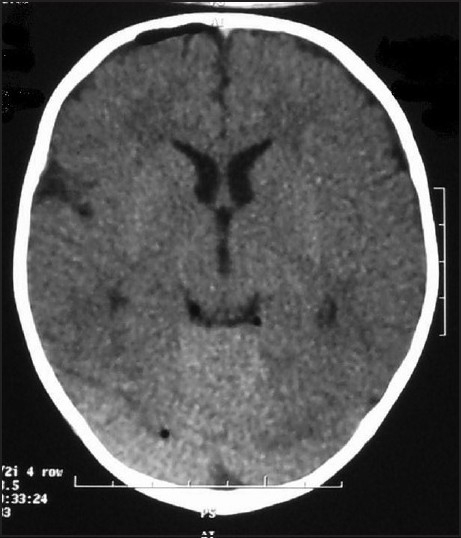
Brain computed tomography scan of the second patient shows a small rim of pneumocephalus

During surgery, no exogenous material entered the field and there was no antibiotic in the water for irrigation. Both patients were extubated in the operation room and transferred to the ward after uneventful recovery. The first patient developed generalized tonic clonic (GTC) seizure 2 hours after surgery, which was controlled with diazepam. The other had one episode of GTC seizure 12 hours after surgery, which was repeated on the next morning. Both attacks were controlled with phenytoin. Laboratory tests and serum electrolytes were normal in both patients. A brain computed tomography (CT) scan was performed in both, which was normal in the first case but revealed a small pneumocephalus of <1 cm thickness in the right frontal area in the second patient. There was no midline shift, edema or hematoma [Figure 1]. Electroencephalography was performed in both patients, which was normal.

Both patients were discharged from the hospital 7 days later and no episodes of seizure occurred afterwards.

## Discussions

The occurrence of seizure after laminectomy for the spinal procedure is very rare. Two cases of such complications have been reported after cervical and thoracic laminectomy,[1,2] although seizure after laminectomy for lipomyelomeningocele has not been reported beforehand.

In myelomeningocele, which is a distinct entity, the range of seizure incidence varies from 14.7% to 29%.[3] Seizure in these patients is mainly related to the hydrocephalus, in particular, to the ventriculoperitoneal shunt and its complications.[3,4] Considering the fact that these patients had lipomyelomeningocele, which never associated with hydrocephalus, the seizure cannot be attributed to this issue.

Different aspects related to the anesthetic course may cause postoperative seizure. However, anesthesia was managed without any hypotensive or hypoxic problems in these patients, with none of the agents being known to be epileptogenic.

Some cerebral and spinal mechanisms can be considered for a justification of the seizure after lipomyelomeningocele surgery. Pneumocephalus has been reported as a reason for generalized convulsion after cervical laminectomy,[2] as can be considered in one of these cases. Postoperative seizure after laminectomy may occur as a result of unrecognized dural tear, resulting in acute cerebrospinal fluid (CSF) loss and the drastic decrease in the CSF pressure.[1] Brain CT in such intracranial hypotension may show dural enhancement or meningeal reaction as decreased distance between the brain and the dura.[1] Different kinds of intracranial hemorrhage, particularly subdural, subsequent to CSF leakage after spinal procedures have been associated with seizure.[1,5]

Some spinal basis can be supposed for this postoperative seizure. The issue of spinal-induced seizure is well documented in the literature and commonly used for experimental purposes.[6,7] The investigations on vagus nerve stimulation on induced spinal cord seizure confirm the seizure with a merely spinal origin.[8] Neurons of the spinal cord, just as in the cerebral cortex, are known to become hyperactive and cause spinal cord “seizures.”[9] Transection of the spinal cord of the cat at a thoracic or lumbar level results in an altered excitability that repeated natural stimulation of the dermatome just caudal to the transection site will induce seizure discharges.[7] Spinal seizure has been reported in humans with transverse myelopathy as well.[10] Although our patients had no paresis before surgery and no cord injury happened during the procedure, it is possible that minor surgical manipulation had stimulated the neural pathways to act as a trigger zone for spinal seizure.

Spinal cord seizure can be induced with stimulation via numerous drugs and chemicals. Strychnine or tranexamic acid was shown to have such influences.[9] Topical or systemic administration of a toxic amount of penicillin can induce convulsive activity.[6] A case of postlaminectomy convulsion due to a large amount of iodophendylate used for preoperative myelogram has been reported as well.[11]

Regarding the fact that chemical spinal stimulation can trigger seizure and that no exogenous substance entered to our field during surgery, it can be considered that the fat molecules inside the lipoma may act as the source of stimulation. It is possible that these molecules were released from the fat tissue in their original form or even in the form of free radicals as a result of resection or thermal coagulation, and had a contact with exposed placode and cerebral neural structures and induced a trigger zone in the cord or brain.

## Conclusion

Although it cannot be determined by certainty, postsurgical seizure after lipomyelomeningocele operation in these cases can be attributed to either pneumocephalus or direct spinal stimulation through surgical manipulation or the potential toxic effect of fat molecules.
